# Attitudes towards assisted dying are influenced by question wording and order: a survey experiment

**DOI:** 10.1186/s12910-016-0107-3

**Published:** 2016-04-27

**Authors:** Morten Magelssen, Magne Supphellen, Per Nortvedt, Lars Johan Materstvedt

**Affiliations:** Centre for Medical Ethics, Institute of Health and Society, University of Oslo, Oslo, Norway; Department of Strategy and Management, Norwegian School of Economics, Bergen, Norway; Department of Philosophy and Religious Studies, Faculty of Humanities, Norwegian University of Science and Technology (NTNU), Trondheim, Norway

**Keywords:** Assisted dying, Euthanasia, Opinion poll, Physician-assisted suicide, Survey experiment

## Abstract

**Background:**

Surveys on attitudes towards assisted dying play an important role in informing public debate, policy and legislation. Unfortunately, surveys are often designed with insufficient attention to framing effects; that is, effects on the respondents’ stated attitudes caused by question wording and context. The purpose of this study was to demonstrate and measure such framing effects.

**Methods:**

Survey experiment in which an eight-question survey on attitudes towards assisted dying was distributed to Norwegian citizens through a web-based panel. Two variations of question wording as well as two variations of question order were employed. Respondents were randomized to receive one of four questionnaire versions.

**Results:**

Three thousand and fifty responses were received. There were moderate to large question wording and question order effects. A majority of Norwegian citizens favour the legalization of assisted dying for patients with terminal or chronic disease.

**Conclusions:**

Stakeholders in the assisted dying debate need to acknowledge potential framing effects, and accordingly should interpret survey results with caution. The same holds for researchers who conduct attitude surveys in the field of bioethics.

**Electronic supplementary material:**

The online version of this article (doi:10.1186/s12910-016-0107-3) contains supplementary material, which is available to authorized users.

## Background

The possible legalization of assisted dying – henceforth AD, which encompasses euthanasia (E), physician-assisted suicide (PAS), and assisted suicide (AS) – is an issue which creates clinical, ethical and political controversy in many Western countries. The Netherlands, Belgium and Luxembourg have legalised E and PAS for both somatic and psychiatric suffering, and there is no requirement of terminal illness [1]. Switzerland and Germany have laws that allow AS but prohibit E. The US states Oregon, Washington, Montana, Vermont and California have legalised PAS, however only for the terminally ill [2].

Due to the logical cleavage between facts and norms, descriptive surveys of attitudes towards AD cannot by their very nature settle the normative question of whether or not AD *ought* to be legalized. Still, such surveys of health professionals and the general public play a pivotal role in informing public debate and policy. That said, researchers face particular challenges when performing research with this field, which include the following: First, medical end-of-life decision making is an extraordinarily *complex* activity. Complexity is further compounded by a great variety in terms, definitions and distinctions used in the clinical, ethical and juridical context as well as within the research itself. Second, the field is *normatively charged*, and partisans often champion their own preferred set of terms, labels and distinctions [3]. This phenomenon is also seen in AD laws; for instance, Oregon’s PAS law is called the “Death with Dignity Act”, avoiding direct reference to PAS and AD. Nor does the text of the law make use of any of these terms.

Both the complexity and the normative charge sometimes cause confusion and misunderstanding among citizens, journalists and politicians, as well as among healthcare professionals. In the literature it is, unfortunately, uncommon for attitude surveys to take these issues sufficiently into account. Instead of stating detailed and unequivocal definitions of key concepts and using normatively balanced questions, questionnaires often employ euphemisms and leave crucial terms undefined [4].

The following definitions correspond to the Dutch definitions [1] and were used in a previous Norwegian study [4]: *Euthanasia* is a doctor intentionally killing a person by injecting drugs, at that person’s voluntary and competent request; *Physician-assisted suicide* is a doctor intentionally helping a person to commit suicide by providing drugs for self-administration, at that person’s voluntary and competent request. These definitions were employed, in slightly edited forms, in the first version of the present study’s questionnaire.

In Norway, the penal code prohibits AD and it is very rarely illegally practiced [5]. There have been several surveys of the attitudes towards legalization in the general population; these are summarized by Nordstrand et al. [4], who criticise these surveys for biased question wording and other fundamental flaws. Although different studies attempt to measure attitudes towards the same practices, namely the different kinds of AD, these practices are presented in quite different ways. In a recent survey the questions exemplified AD by introducing the case of a particular, suffering patient who was portrayed in ways that engage the respondent’s sympathy [6]. By contrast, Nordstrand et al.’s questions cited the Dutch definitions without invoking particular patient stories, and used the rather blunt terms “suicide” and “killing”, which may be off-putting to some respondents [4]. Our hypothesis is that differences such as these will influence respondents, inclining them to assent *more* in the former case, *less* in the latter, to the propositions about the legalization of AD.

Indeed, such *framing effects* of questionnaire design on responses are well known from the field of social psychology in which they have been extensively documented [7]. According to Chong and Druckman’s definition, framing effects “occur when (often small) changes in the presentation of an issue or an event produce (sometimes large) changes of opinion” [8]. As they explain,

The major premise of framing theory is that an issue can be viewed from a variety of perspectives and be construed as having implications for multiple values or considerations. Framing refers to the process by which people develop a particular conceptualization of an issue or reorient their thinking about an issue [8].

Two important subtypes of framing effects are wording and context effects, respectively. As regards *wording effects*, answers are influenced by the precise wording of the attitude question. Specific terms may give rise to particular interpretations or evoke particular associations and feelings in the respondent. For instance, in a classic study carried out in the USA, 23 % of the public agreed that too little was being spent on “welfare”, whereas 64 % agreed that too little was being spent on “assistance to the poor” [9]. The two terms were intended to describe the same policy, yet evidently evoked different judgments in the minds of respondents.

In *context effects*, answers are influenced by the context in which an attitude question is asked. Such a context may include, for instance, the nature of the preceding questions or attributes of the interviewer or organization that presents the questionnaire. An important kind of context effect is the *question order effect*, in which question order influences the way the target question is interpreted and answered. Depending on the direction of the influence, the question order effect may be either an *assimilation effect* or a *contrast effect* [10]*.* Assimilation effects entail that question order reduces differences in responses between adjacent questions; in contrast effects, question order increases differences. Question order effects occur when the thoughts and feelings triggered by a question carry over to the next question, thereby influencing the response.

Surveys of AD typically include a series of questions addressing different aspects of the phenomenon. Some issues are more controversial than others, such as the proposal that AD should be offered also for mental illness or tiredness of life. Would it matter if such controversial questions were put first (or last) in a survey? To our knowledge, such question order effects have not been addressed in previous research on AD.

When framing effects are exploited in surveys in order to shed light on substantive or methodological issues, this is termed a *survey experiment* [11, 12]. But framing effects and survey experiments have received surprisingly little attention within bioethics and biopolitics. This is particularly striking given how fond the media are of surveys of health professionals’ and the public’s attitudes towards pressing controversies in bioethics – and in view of how often the results of such polls are invoked as arguments in the public debate.

We have indentified three studies that examine framing effects in surveys on AD. However, none of these studies are survey experiments proper in which respondents are randomized into groups receiving alternative questionnaires.

In a Canadian study, 991 respondents from the general population were asked to take a stand on euthanasia based on two different descriptions of the act, none of which used the term euthanasia [13]. The first question portrayed euthanasia in a more positive light, by emphasizing the patient’s short life-expectancy and great suffering. Here, 80.8 % agreed that the doctor “should be allowed by law to end the patient’s life through mercy killing”; available alternatives were “yes”, “no” and “don’t know”. The second question presented both the act and the circumstances in a less positive way, in response to which 69.6 % found it “completely” or “somewhat acceptable” that the doctor would “give an injection which causes death”. Results do indicate a wording effect; however, not only the description of euthanasia but also response alternatives were different. The latter may be partly responsible for the impact observed. In addition, because all respondents were posed both questions in the same order, question ordering may have influenced answers. The study also found that there is considerable confusion with regard to the terminology; 20 % were unable to identify a description of an act of euthanasia with the correct term. Among those unable to distinguish euthanasia from withdrawal or withholding of life-prolonging treatment, a higher proportion accepted euthanasia.

A Swedish study showed differences in rates of acceptance of euthanasia when question introduction, wording and answer categories were varied [14]. Respondents received one of two questionnaire variants. Nevertheless, these findings are of limited value since the study was conducted as two separate studies and therefore not randomized.

An Australian study investigated patients’ views on AD with the aid of face-to-face interviews in which all respondents were asked a set of questions describing AD in different ways [15]. The study demonstrated that question wording impacted on answers. In particular, to the question “Do you support the idea of euthanasia?” 79 % answered yes; 70 % answered yes to “Do you believe that a doctor should be able to assist a patient to die?”; and only 34 % gave an affirmative answer to “Do you believe that a doctor should be able to deliberately bring about a patient’s death?”.

The purpose of the present study was to explore framing effects in the setting of surveys on AD by way of a survey experiment. Our hypothesis was that both wording and order effects would be present and of significant size.

## Methods

As part of the Norwegian Bioethics Attitude Survey (NOBAS),^1^ a web-based questionnaire was distributed by the commercial firm Respons Analyse to a sample of the general public in June 2015. Respondents were recruited from Respons Analyse’s nationally representative web-based panel of respondents in four successive waves until the target number of respondents was reached. In the final waves, respondents were selected with a view to demographical balancing. A total of 22,660 respondents were invited and informed about the nature of the survey by email. Participation was voluntary and completion of the questionnaire was considered as implicit consent. Responses were anonymous. All questions had to be answered for the response to be registered. 3050 completed questionnaires were returned by respondents, a response rate of 13.5 %. 101 respondents commenced the questionnaire without finishing it; data from these respondents have not been included. The analyses were performed on weighted data (Table 1). Specifically, females, younger respondents (<35 years) and respondents from certain geographical areas were underrepresented in the sample, as compared with the population profile of the entire country. Data from these groups were given extra weight in the estimation of mean scores on study variables (Table 1).

**Table 1:** Demographic characteristics of respondents

Characteristic		N (unweighted)	% (unweighted)	% (weighted)
Gender	Female	1480	48.5	50.4
	Male	1570	51.5	49.6
Age	16–24	129	4.2	14.9
	25–34	443	14.5	16.3
	35–44	582	19.1	18.7
	45–54	642	21.0	17.1
	55+	1254	41.1	33.1
Level of education	Primary school	133	4.4	5.0
	Upper secondary school	756	24.8	26.4
	College/university ≤3 years	858	28.1	28.7
	College/university >3 years	1275	41.8	38.9
	Unanswered	28	0.9	0.9
Religious belief	Non-religious	1482	48.6	50.2
	Christian	1241	40.7	39.4
	Muslim	15	0.5	0.5
	Other religions	88	2.9	2.9
	Unanswered	224	7.3	7.1

We used a two (question wording) by two (question order) factorial between-subjects design to test our predictions. Respondents were randomized into four equal groups who received one of the four versions of the questionnaire.

The full questionnaires translated into English may be accessed in Additional file 1. The two versions on question wording were designed in line with questions used in previous surveys in Norway [4, 6]; we have labelled these the “concept-focused” (version 1) and “context-focused” (version 2) versions, respectively. The most widely used Norwegian term corresponding to AD, “aktiv dødshjelp”, which literally means “active aid in dying”, was employed throughout. The introduction to the questionnaire on AD differed between the two versions. Version 1 stated definitions of E and PAS corresponding to the definitions given in the introduction. Version 2 did not define, nor mention, the very terms E/PAS, but stated simply that “Active aid in dying is also called ’self-determined ending of life’”, a formulation frequently used by a Norwegian pro-AD organization.^2^ Both introductions went on to state that AD is illegal in Norway, subsequently listing the European countries where it is legal. Both introductions contained the same definition of non-treatment decisions (NTDs): “ending (or not starting) life-prolonging treatment for a dying patient”. In Norwegian, the term that most closely resembles NTDs is “behandlingsbegrensning” – literally “treatment limitation”.

Both questionnaire versions consisted of eight questions (Tables 2 and 3). In both versions the first three questions were intended to describe the same medical practices (PAS for terminal disease, E for terminal disease, and AD for chronic disease, in this order of appearance), yet with quite differently worded questions. The final five questions were identical across the two questionnaire versions. Answers to all questions were given on a five point scale (strongly agree – somewhat agree – neither agree or disagree – somewhat disagree – strongly disagree). “Do not know” was not an option. To proceed, respondents were required to give an answer; thus, all registered respondents answered all questions. Subsequent sections asked about attitudes towards other bioethical questions, and collected demographic information.

**Table 2:** Questions on assisted dying (AD)

Question no.	Questionnaire version 1 – concept-focused	Questionnaire version 2 – context-focused	Intended to describe
1	Physician-assisted suicide should be allowed for persons who have a terminal illness with short life expectancy	A dying patient is in great pain. To what degreee are you in agreement or disagreement with the statement that a doctor, after careful consideration, and upon the patient’s request, should be allowed to prescribe a lethal drug dose that the patient can choose to take to avoid great suffering?	PAS for terminal illness
2	Euthanasia should be allowed for persons who have a terminal illness with short life expectancy	Suppose the dying patient with great pain is so ill that he or she is unable to swallow the lethal drug. To what degreee are you in agreement or disagreement with the statement that a doctor, after careful consideration, and upon the patient’s request, should be allowed to administer a lethal injection?	E for terminal illness
3	Active aid in dying should also be allowed for persons who have an incurable chronic disease but who are not dying	A patient is incurably ill but not dying, and experiences great suffering that cannot be alleviated sufficiently. To what degree are you in agreement or disagreement with the statement that a doctor, after careful consideration, and if the patient requests it, should be allowed to provide active aid in dying?	AD for chronic disease

**Table 3:** Questions on assisted dying (AD)

Question no.	Questionnaire versions 1 & 2
4	Active aid in dying should be allowed for persons who have a mental illness
5	Active aid in dying should be allowed also for persons who do not suffer from serious illness, but who are tired of life and want to die
6	The legalization of active aid in dying may result in weak groups experiencing pressure to request aid in dying
7	Instead of allowing active aid in dying, we should develop and expand the provision of palliative care to the dying
8	Treatment limitation can sometimes be the right decision, to avoid a distressing prolongation of the dying process

Two different question orders were used. The first version followed that of Tables 2 and 3, starting with question Q1 concerning AD for those terminally ill and with short life expectancy, the archetypical case in the debate on AD. In the second version, participants answered Q4-8 before Q1-3. Question 4 and 5 explore opinions on AD in cases of mental illness and tiredness of life. AD was assumed to be more controversial in these situations [4, 16]. We predicted that if one starts by asking more controversial questions, this will alter responses to less controversial questions on AD.

### Questionnaire validation

Several steps were taken to validate the questionnaire. Key questions were adopted from previous studies [4, 6]. Several experts on medical ethics and survey methodology gave input to both choice of measures and question wording. Two lay persons and two survey experts, all blind to the purpose of the study, gave feedback on an earlier version of the questionnaire. This test lead to the removal of three questions (due to respondent fatigue), and also to minor changes in the wording of two particular questions.

### Statistical analyses

In the analyses we used a five-point Likert scale with “Strongly disagree” (=1) to “Strongly agree” (=5) as scale anchors for all scaled variables. Responses were analysed using IBM SPSS Statistics version 22. MANOVA (multivariate analysis of variance) was used to test differences between questionnaires and demographic subgroups. Three tests were used for the MANOVAs: Wilk’s Lambda, Pillai’s Trace, and Hotelling’s Trace. F-tests were used for follow-up testing of group differences for each question. P-values are adjusted for multiple comparisons. All tests were two-tailed.

## Results

### Attitudes towards assisted dying (AD)

Table 4 shows mean answers to Q1-8. Distinct majorities hold a positive attitude towards legalization of PAS (Q1) and, slightly less, euthanasia (Q2). A small majority also favours AD for patients who suffer from a chronic disease (Q3). Respondents were critical, however, of AD for mental illness (Q4) or tiredness of life (Q5). The statement that legalization of AD could put pressure on vulnerable groups (Q6) was supported by a majority. The expansion of palliative care received strong support (Q7), as did the practice of occasionally limiting life-prolonging treatment towards the end of life (NTDs; Q8).

**Table 4:** Tests of question wording and question order effects for eight questions about AD (Means from MANOVA; SD in parenthesis)

Variables		Q1 (PAS for terminal disease)	Q2 (E for terminal disease)	Q3 (AD for chronic disease)	Q4 (AD for mental illness)	Q5 (AD for tiredness of life)	Q6 (pressure on weak groups)	Q7 (palliative care)	Q8 (NTDs)
Total		3.94 (1.32)	3.70 (1.41)	3.25 (1.46)	2.14 (1.28)	2.02 (1.31)	3.27 (1.35)	3.61 (1.23)	4.29 (0.98)
Question wording	Concept-focused	3.78 (1.29)	3.63 (1.40)	2.84 (1.42)	2.02 (1.22)	1.88 (1.22)	3.23 (1.38)	3.66 (1.19)	4.25 (1.00)
	Context-focused	4.11*** (1.24)	3.77** (1.40)	3.67*** (1.39)	2.27*** (1.33)	2.16*** (1.38)	3.30 (1.32)	3.56* (1.26)	4.33* (0.95)
Question order	Less controversial first (Q1-Q3)	3.85 (1.33)	3.65 (1.41)	3.03 (1.45)	2.03 (1.21)	1.82 (1.18)	3.17 (1.38)	3.61 (1.22)	4.25 (1.01)
	Most controversial first (Q4-Q5)	4.03*** (1.29)	3.75* (1.41)	3.47*** (1.44)	2.26*** (1.34)	2.21*** (1.39)	3.36*** (1.31)	3.61 (1.25)	4.32* (0.95)

Note: Numbers are mean scores on a five-point Likert scale: 1 = strongly disagree; 5 = strongly agree (SDs in parenthesis). The MANOVA test of group differences was significant for both question wording (Pillai’s trace = .103; Wilk’s Lambda = .897; Hotelling’s trace = .115; *F* = 49.2; p-values for all three tests < .000) and for question order (Pillai’s trace = .0.25; Wilk’s Lambda = .975; Hotelling’s trace = .25; *F* = 10.8; p-values for all three tests < .000). The p-values in the Table were generated from follow-up F-test of mean differences between experimental groups for each question.

*: *p* < 0.05; **: *p* < 0.01; ***: *p* < 0.001. All tests are two-tailed

### Correlation with demographic variables

Responses were correlated with demographic variables (Table 5). The MANOVA tests for age groups, gender, education level, and religious group were all significant with F-values in the range of 6.78–15.46 and p-values < .001.

**Table 5:** Comparison of means across demographic subgroups (MANOVA; SD in parenthesis)

Variables		Q1 (PAS)	Q2 (E)	Q3 (AD for chronic disease)
Age groups	16–24	4.09 (1.12)	3.83 (1.28)	3.51 (1.27)
	25–34	4.01 (1.21)	3.70 (1.35)	3.38 (1.40)
	35–44	4.11 (1.21)	3.89 (1.30)	3.38 (1.42)
	45–54	3.95 (1.37)	3.70 (1.46)	3.22 (1.51)
	55+	3.76^a^ (1.43)	3.55^b^ (1.32)	3.04^a^ (1.53)
Gender	Male	3.98 (1.29)	3.75 (1.37)	3.33 (1.44)
	Female	3.91 (1.34)	3.65^c^ (1.44)	3.17^d^ (1.48)
Educational levels	Primary school	4.08 (1.16)	3.76 (1.30)	3.36 (1.38)
	Upper secondary school	4.18 (1.14)	3.98 1.23)	3.45 (1.42)
	≤3 years higher ed.	3.97 (1.29)	3.75 (1.39)	3.32 (1.44)
	>3 years higher ed.	3.76^e^ (1.42)	3.49^f^ (1.51)	3.06^f^ (1.50)
Religious beliefs	No religion	4.23 (1.08)	3.99 (1.23)	3.60 (1.34)
	Christian	3.62^g^ (1.48)	3.38^g^ (1.53)	2.85^g^ (1.52)
	Muslim	3.06^g^ (1.34)	3.00^g^ (1.55)	2.69^g^ (1.45)
	Other religions	4.20 (1.20)	3.97 (1.37)	3.47 (1.45)

Note: Numbers are mean scores on a five-point Likert scale: 1 = completely disagree; 5 = completely agree (SDs in parenthesis). Three tests were used for the MANOVAs: Wilk’s Lambda, Pillai’s Trace, and Hotelling’s Trace. MANOVA tests for age groups, gender, education level, and religious group were all significant with F-values in the range of 6.78–15.46 and p-values < .000. F-tests were used for follow-up testing of group differences for each question. All tests were two-tailed

^a^mean in the oldest group significantly lower than the mean in all other groups, except age group 45–54 (*p* < 0.001)

^b^mean in the oldest group significantly lower than the mean in the two youngest age groups (16–24 and 25–34; *p* < 0.001)

^c^females score significantly lower than males (*p* < 0.05)

^d^females score significantly lower than males (*p* < 0.01)

^e^mean in this group significantly lower than the mean in all other groups (*p* < 0.001)

^f^mean in this group significantly lower than the mean in all other groups, except the group with only primary school education (*p* < 0.001)

^g^mean for Christians and Muslim are significantly lower than for No religion and Other religions (*p* < 0.001). Means for Christians and Muslims are not significantly different

Table 5 displays the clear tendency of more positive attitudes towards AD in the younger age categories. The three youngest groups (16–24, 25–34, and 35–44 years) score significantly higher than the oldest group (55+) on Q1 (PAS) and Q3 (AD for chronic disease). The two youngest groups (16–24 and 25–34) also score significantly higher on Q2 (E) than the oldest group (55+). As regards the impact of education, we observe that the group with the highest education level (>3 years of higher education) score significantly lower on Q1 (PAS) than the other groups with lower education. For Q2 (E) and Q3 (AD for chronic disease), the group with the highest education also scores significantly lower than other groups, with the exception of the group with the lowest education level (only primary school).

On questions Q1-Q3, Muslims and Christians score significantly lower than respondents in the No Religion or Other Religion categories. There are also gender differences, in that mean scores for males are significantly higher on Q2 (E) and Q3 (AD for chronic disease) than for females.

### Wording and order effects

Table 4 shows the MANOVA tests of question wording and question order. We only show main effects of question wording and question order, respectively, because the interaction was non-significant. The MANOVA tests for question wording were all significant (Pillai’s trace = .103; Wilk’s Lambda = .897; Hotelling’s trace = .115; *F* = 49.2; p-values for all three tests < .001). The results of follow-up tests for each question are reported in Table 4. There are substantial question wording effects. Mean scores are significantly higher for the context-focused (version 2) than the concept-focused (version 1) wording on all the questions about attitudes towards legalization of AD (Q1-5). The wording effect is particularly strong on the question about AD for chronic disease. Here the mean score in the contextual wording version is 29 % higher than in the concept-focused version (3.67 vs. 2.84 respectively).

The MANOVA tests for question order were also significant (Pillai’s trace = .0.25; Wilk’s Lambda = .975; Hotelling’s trace = .25; F =10.8; p-values for all three tests < .001). The follow-up tests of question order for each question identified clear signs of contrast effects (Table 4). When markedly controversial questions were asked first (Q4-5), scores on less controversial questions (Q1-3) were significantly higher. For example, participants who initially indicated a less positive attitude towards AD for tiredness of life (Q5, for which the mean is close to 2 (i.e., “somewhat disagree”) on the 5-point scale), subsequently indicated more positive attitudes towards AD for terminal (Q1-2), and, in particular, chronic disease (Q3) than without the more controversial preceding question. Thus, a negative attitude towards initial, controversial questions seemed to trigger more positive responses to less controversial issues. Correspondingly, when the less controversial questions (Q1-3) were asked before the more controversial (Q4-5), scores on controversial questions were lower (less accepting). Apparently, sharing positive attitudes towards AD concerning an archetypical situation such as the terminal patient with short life expectancy triggered a need to be more restrictive in responses to more controversial situations.

## Discussion

### Explaining question framing effects

This study demonstrates the presence of question wording and order effects in the context of a survey of attitudes towards the legalization of AD. These effects were not only statistically significant, but also in most cases sufficiently large to be of considerable practical importance.

As predicted, the contextual version (#2) of the questionnaire produced greater assent to the legalization of AD than did the concept-focused version (#1). In contrast to the latter version, the contextual version evokes an image of a particular patient portrayed in ways that engage the respondent’s sympathy (e.g., “in great pain”), while at the same time underscoring the rationale for AD (“avoid great suffering”) and reassuring that decisions will not be taken lightly (“thorough evaluation”). The introduction to the contextual version also describes AD in a way that is likely to have positive connotations, namely as “self-determined ending of life”, and avoids the concept-focused version’s terms “intentional killing” and “aid in a person’s suicide” and the negative connotations associated therewith. On our interpretation, these features are most likely responsible for the wording effects observed. Notably, the effect carries over to the subsequent questions; assent to the proposals of AD for mental illness (Q4) and tiredness of life (Q5) is significantly higher in the contextual version.

The variations in question order exhibited a *contrast effect*, with higher assent to proposals to legalize AD for terminal and chronic disease (Q1-3) when the most controversial proposals (Q4-5) were presented first. The suggestion that AD could be offered even for individuals with mental illness or people who are merely tired of life appearently made the proposal to legalize AD for terminal and chronic disease less controversial and more socially acceptable. Rejecting the former controversial proposals (Q4-5) would mean that the respondent could still accept the latter (Q1-3) and yet perceive their own position as nuanced, avoiding both extremes. Similarly, the respondent who was first exposed to the less controversial questions Q1-3 might have experienced a need to distance him- or herself from the more extreme proposals of Q4-5.

### Question framing effects are of practical importance

We find the size of the wording and order effects to be sufficiently large to be important for policy formation and public debate, as well as for attitude research. First, it is well known to policy makers and activists that framing the issue in carefully chosen terms and colouring it with evocative metaphors, is instrumental in shaping the public’s views [17]. The present study demonstrates such framing effects for the topic of AD, and suggests that effect sizes are rather substantial. From this it follows that proponents of AD are likely to win more support for their cause if they portray actual, suffering patients and by invoking the normative language of self-determination. In contrast, opponents of AD appear to benefit from using the Dutch AD definitions which include the terms “killing” and “suicide”, as well as discussing the topic detached from stories of individual patients in extreme circumstances.

A further interpretation is that AD proponents would most likely benefit from sharply demarcating their own proposal for legislation from more extreme proposals that would include, for instance, patients with mental illnesses or those who suffer from (mere) tiredness of life. AD proponents may thus be able to invoke contrast effects deliberately: by explicitly rejecting the more extreme AD proposals, their own position appears more nuanced and responsible. For the camp of AD opponents, an option which presents itself is trying to undermine the contrast effect by, for instance, portraying the assent to AD even in a carefully circumscribed set of cases as merely a first step onto an inevitably slippery slope.

Second, in demonstrating that framing and order effects are of significant size, the present study can be read as an implicit critique of most previous attitude surveys on AD. There is no denying that many surveys are quite naïve and simplistic in their presentation of the issue, and often also in using just one or two questions and/or constraining or forcing respondents into answering either “yes” or “no”, thereby missing out on nuances and ambivalence among respondents.

We believe that more attitude research in bioethics should be done by way of survey experiments. Such study designs enable multiple perspectives on the topic in question within the same study. There are, however, two drawbacks with such designs that should be mentioned: firstly, study design becomes more complex; secondly, large sample sizes, as in the present survey, are needed to achieve statistical power.

What does the apparent malleability of people’s attitudes towards AD disclose about these attitudes? First of all, the presence of significant framing effects is not unique to the issue of AD; such effects have been demonstrated on a wide range of topics [8]. It is debated whether or not people’s susceptibility to framing is a good or a bad thing [8]. On the one hand, the stability, depth and consistency of people’s attitudes may be questioned in light of large framing effects; can such attitudes really be said to be *informed* and well thought through, and if not, should they have any political significance? On the other hand, susceptibility to framing shows that respondents are sensitive to arguments and context; such sensitivity is an important human capacity as well as key in democratic processes. The significant framing effects may also indicate that large portions of the public have not engaged thoroughly with the issue of AD. As Chong and Druckman state, “Theoretically, we expect that framing effects diminish with active engagement with issues. In particular, biased representations of issues should be less influential as citizens become exposed to the full array of alternative arguments” [8]. In light of this contention, an hypothesis worth exploring in future research is that groups such as health professionals, politicians and professional bioethicists are *less* susceptible to framing effects than the general public.

### Attitudes towards AD among the Norwegian public

Answers to the four questionnaire versions cannot be straightforwardly pooled and taken to represent Norwegians’ views on AD. If pooled, it must be kept in mind that the resulting average scores stem from questionnaires with significant differences. Notwithstanding this caveat, however, we would argue that exposing respondents to different ways of posing the key questions constitutes a kind of method triangulation in which the question at hand – what are the public’s views on AD? – is perceived from several angles; arguably, a more detailed picture of those views then emerges than if a single method was employed. In a similar vein, quantitative research on opinions on AD can and should be complemented by qualitative research, which has the potential for enriching the account of such attitudes with depth and complexity [18, 19].

If we proceed to combine the results on the four questionnaire versions, keeping in mind the way in which the results have arisen, it appears that majorities support the legalization of AD for terminal and chronic disease, ranging from a preponderant majority of 75.8 % for PAS for terminal disease to a slight majority of 51.4 % for AD for chronic disease. Few support AD in other situations. Because previous Norwegian studies differ radically from ours and typically are flawed in important respects (e.g., only one or a few questions, key terms undefined, biased question wording) [4], our results are not directly comparable. Our finding that the legalization of AD is supported by a majority is however in line with previous surveys.

Most attitude surveys carried out in other Western countries show that majorities support AD, including in most countries where AD is presently illegal [16]. Our findings reveal that the level of support among Norwegians varies across demographic subgroups in a way partly different from what has been shown to be the case in research in other countries. We find that the respondents with the highest level of education (>3 years of higher education) were less accepting of PAS than groups with lower levels of education. For questions on euthanasia and AD for chronic disease, the higher educated were also less accepting than respondents with a medium level of education, but not significantly different from the group with the least education. The finding that those most educated tend to be less accepting contradicts the tendency in a recent survey on 15 other countries [16].

We also observe that the younger groups (16–24, 25–34, and 35–44) were more likely to support AD than the oldest group (55+). This may indicate a cohort effect in which support for AD will increase as time goes by. The finding that Christian and Muslim respondents were less positive towards AD than the non-religious, was expected. However, we note that there is still considerable support for AD among Christians (Table 5; e.g., mean score 3.62 on Q1).

### Potential limitations

The study design does not enable separate assessment of the effect of the different questionnaire introductions on the one hand, and of the different question wording on the other: the effects measured are for introductions and questions combined.

There is a difference between the concept- and the context-focused questionnaire versions in that only the latter mentions pain and suffering (cf. Q1-3, Tables 2 and 3). This difference, some might argue, is substantial and does not only involve wording effects. However, we do not agree with this objection. The context-focused version’s portrayal of PAS/E (Q1-2) involves a dying patient in great pain, yet this is precisely the kind of patient for whom a majority of the public would want PAS/E to be available, as research has repeatedly demonstrated. In that sense it is a paradigmatic example, and thus constitutes the background against which many respondents will view the concept-focused questionnaire version too. We accordingly find the propositions to be sufficently equivalent across the two questionnaire versions: They portray the legalization of PAS or E for terminal illness, or AD for chronic disease, respectively.

The response rate is very low at 13.5 %, raising the issue of a possible non-response bias. However, the phenomenon of low and declining response rates is a problem that affects population surveys all over the Western world [20]. Much effort has been put into studying consequences of nonresponse for study validity. Detailed analyses indicate that surveys may well be representative in spite of very low response rates [21, 22]. Furthermore, surveys of respondent attitudes, such as the present, are less at risk for nonresponse bias than surveys of respondent activities [21]. Our survey has attempted to mitigate nonresponse bias through selective invitations to balance respondents on demographic paramenters. Still, we cannot rule out that our results are influenced by a nonresponse bias, for instance in that respondents who are less interested in bioethical issues might have been less likely to complete the survey.

## Conclusion

The present study demonstrates significant question framing and order effects in a survey on attitudes towards AD. Journalists, politicians and others should be aware of such effects and interpret survey results accordingly, as should researchers who conduct attitude surveys in bioethics. Designs as per the principles of survey experiments may enrich the yield of such surveys.

## Ethics approval

Due to responses being anonymous formal ethical approval was not required according to Norwegian regulation.

## Availability of data and materials

The data used in this article are not available for sharing as they will be used for subsequent scientific analyses.

## Additional file

Additional file 1: NOBAS 2015 – questionnaire on assisted dying. Translated from Norwegian to English by the authors. (DOCX 98 kb)

## Abbreviations

AD: assisted dying
E: euthanasia
MANOVA: multivariate analysis of variance
NOBAS: Norwegian Bioethics Attitude Survey
NTD: non-treatment decision
PAS: physician-assisted suicide

## Footnotes

1 For further information about the NOBAS project, see http://www.med.uio.no/helsam/english/research/projects/norwegian-bioethics-attitude-survey/index.html.

2 Foreningen Retten til en verdig død, www.livstestament.org. Accessed 17 Dec 2015.
