# Supplementary material for: Attitudes towards assisted dying are influenced by question wording and order: a survey experiment
Source: BMC Med Ethics. 2016 Apr 27;17:24. doi: 10.1186/s12910-016-0107-3 (PMC4848799; doi:10.1186/s12910-016-0107-3)
Supplement: Additional file 1: — NOBAS 2015 – questionnaire on assisted dying. Translated from Norwegian to English by the authors. (DOCX 98 kb) [file 12910_2016_107_MOESM1_ESM.docx]

**For online appendix: NOBAS 2015 – questionnaire on assisted dying. Translated from Norwegian to English by the authors.**

Answers to all questions were given on a five point scale (strongly agree – somewhat agree – neither agree or disagree – somewhat disagree – strongly disagree). ”Do not know” was not an option.

**First version (descriptive):**

Active aid in dying is a collective term for euthanasia and physician-assisted suicide. *Euthanasia* is a doctor’s intentional killing of a person by injecting lethal drugs, at the person’s voluntary request. Correspondingly, *physician-assisted suicide* is a doctor’s aid in a person’s suicide, by providing drugs for the person to self-administer.

In Norway, both forms of active aid in dying are illegal. In Europe, the Netherlands, Belgium and Luxembourg allow both euthanasia and physician-assisted suicide, whereas Switzerland allows assisted suicide.

Ending (or not starting) life-prolonging treatment for a dying patient is not considered aid in dying, but is called *treatment limitation*. Such treatment limitation occurs frequently in the Norwegian health service. Two examples may be refraining from treating an infection in a patient who is close to dying, or to end ventilator treatment for a dying patient. In both cases this takes place according to the wish of the patient.

Consider the following statements:

A. Physician-assisted suicide should be allowed for persons who have a terminal illness with short life expectancy

B. Euthanasia should be allowed for persons who have a terminal illness with short life expectancy

C. Active aid in dying should also be allowed for persons who have an incurable chronic disease but who are not dying

D. Active aid in dying should be allowed for persons who have a mental illness

E. Active aid in dying should be allowed also for persons who do not suffer from serious illness, but who are tired of life and want to die

F. The legalization of active aid in dying may result in weak groups experiencing pressure to request aid in dying

G. Instead of allowing active aid in dying, we should develop and expand the provision of palliative care to the dying

H. Treatment limitation can sometimes be the right decision, to avoid a distressing prolongation of the dying process

**Second version (contextual):**

Active aid in dying is also called ”self-determined ending of life”. In Norway, active aid in dying is illegal. In Europe, the Netherlands, Belgium, Luxembourg and Switzerland allow this in various forms.

Ending (or not starting) life-prolonging treatment for a dying patient is not considered aid in dying, but is called *treatment limitation*. Such treatment limitation occurs frequently in the Norwegian health service. Two examples may be refraining from treating an infection in a patient who is close to dying, or to end ventilator treatment for a dying patient. In both cases this takes place according to the wish of the patient.

Consider the following statements:

A. A dying patient is in great pain. To what degreee are you in agreement or disagreement with the statement that a doctor, after careful consideration, and upon the patient's request, should be allowed to prescribe a lethal drug dose that the patient can choose to take to avoid great suffering?

B. Suppose the dying patient with great pain is so ill that he or she is unable to swallow the lethal drug. To what degreee are you in agreement or disagreement with the statement that a doctor, after careful consideration, and upon the patient's request, should be allowed to administer a lethal injection?

C. A patient is incurably ill but not dying, and experiences great suffering that cannot be alleviated sufficiently. To what degree are you in agreement or disagreement with the statement that a doctor, after careful consideration, and if the patient requests it, should be allowed to provide active aid in dying?

D. Active aid in dying should be allowed for persons who have a mental illness

E. Active aid in dying should be allowed also for persons who do not suffer from serious illness, but who are tired of life and want to die

F. The legalization of active aid in dying may result in weak groups experiencing pressure to request aid in dying

G. Instead of allowing active aid in dying, we should develop and expand the provision of palliative care to the dying

H. Treatment limitation can sometimes be the right decision, to avoid a distressing prolongation of the dying process
